# Internet Services for Communicating With the General Practice: Barely Noticed and Used by Patients

**DOI:** 10.2196/ijmr.4245

**Published:** 2015-11-24

**Authors:** Martine WJ Huygens, Joan Vermeulen, Roland D Friele, Onno CP van Schayck, Judith D de Jong, Luc P de Witte

**Affiliations:** ^1^ School for Public Health and Primary Care (CAPHRI) Department of Health Services Research Maastricht University Maastricht Netherlands; ^2^ Netherlands Institute for Health Services Research (NIVEL) Utrecht Netherlands; ^3^ Tilburg School of Social and Behavioral Sciences Tranzo Tilburg University Tilburg Netherlands; ^4^ School for Public Health and Primary Care (CAPHRI) Department of Family Medicine Maastricht University Maastricht Netherlands; ^5^ Research Center Technology and Care Zuyd University of Applied Sciences Heerlen Netherlands

**Keywords:** eHealth, online communication, primary care, general practice

## Abstract

**Background:**

The Netherlands is one of the frontrunners of eHealth in Europe. Many general practices offer Internet services, which can be used by patients to communicate with their general practice. In promoting and implementing such services, it is important to gain insight into patients’ actual use and intention toward using.

**Objective:**

The objective of the study is to investigate the actual use and intention toward using Internet services to communicate with the general practice by the general practice population. The secondary objective is to study the factors and characteristics that influence their intention to use such services.

**Methods:**

There were 1500 members of the Dutch Health Care Consumer Panel, age over 18 years, that were invited to participate in this cross-sectional study. People who had contacted their general practitioner at least once in the past year were included. Participants were asked to fill out a questionnaire about the following services: Internet appointment planning, asking questions on the Internet, email reminders about appointments, Internet prescription refill requests, Internet access to medical data, and Internet video consultation. Participants indicated whether they had used these services in the past year, they would like to use them, and whether they thought their general practice had these services. For the first two services, participants rated items based on the unified theory of acceptance and use of technology complemented with additional constructs. These items were divided into six subscales: effort expectancy, performance expectancy, trust, attitude, facilitating conditions, and social influence.

**Results:**

There were 546 participants that were included in the analyses out of 593 who met the inclusion criteria. The participants had a mean age of 53 years (SD 15.4), 43.6% (n=238) were male, and 66.8% (n=365) had at least one chronic illness. Actual use of the services varied between 0% (n=0, video consultation) and 10.4% (n=57, requesting prescription refill by Internet). The proportion of participants with a positive intention to use the service varied between 14.7% (n=80, video consultation) and 48.7% (n=266, Internet access to medical data). For each service, approximately half indicated that they did not know whether the service was available. Univariate logistic regression analyses revealed that all the constructs as well as age, level of education, and Internet usage had a significant association with intention toward using Internet appointment planning and asking questions by Internet.

**Conclusions:**

Internet communication services to contact the general practice are not yet frequently used by this population. Although a substantial number of persons have a positive intention toward using such services, not all people who receive primary care seem willing to use them. The lack of awareness of the availability and functionality of such services might play an important role.

## Introduction

### Internet Communication Services for Patients in Primary Care

In primary care, there is a growing emphasis on Internet information and communication services (or eHealth) for providing patients with Internet access to the general practice and their medical data. Moving from “traditional care” toward eHealth is a key goal of the European Union. In the digital agenda for Europe, 3 specific actions are stated: widespread deployment of telemedicine, patients’ access to their medical data, and interoperability [1]. The Netherlands, Denmark, Sweden, Finland, and the United Kingdom are frontrunners in the field of eHealth in Europe [2]. Of these frontrunners, the Netherlands leads in the percentage of households with an Internet connection and broadband connection. In addition, the Netherlands has the highest percentage of people who are regular Internet users and who use eGovernment services [2]. A recently published eHealth monitor (a part of which provided the data for this study) describes the development and progress of eHealth in the Netherlands [3]. It reported that 91% of 304 surveyed general practitioners (GPs) offered one or more Internet services to their patients by which they could contact their GP or the general practice. The most frequently offered services were Internet prescription refill requests (66%) and the ability to ask questions via email or websites (56%). In addition, 14% of the GPs indicated that they offered services to plan appointments on the Internet and 25% indicated that they intended to implement this service within 1 year.

The implementation of Internet communication services in primary care is expected to have positive effects because these services can increase the efficiency of care, patient satisfaction, and quality of care [4-8]. For instance, previous research has indicated that the use of an Internet messaging system or the use of email for communication in primary care practice can reduce the number of office visits (but not phone consultations) [4], can improve the communication between health care providers and patients [5,7], and is assessed by patients as convenient, time saving, and useful [6].

### Investigating Internet Services for Patients

Although these results are promising, previous research has shown that these services are not routinely used [9] and not frequently accepted by patients [10]. To predict patients’ willingness to use a service, physical, psychological, and social factors, and the needs of patients, have to be understood [10]. To improve future adoption, the actual use of Internet communication services and the factors that influence the intention to use such services should be investigated.

The technology acceptance model (TAM) [11] is the most well-known and robust model for testing technology acceptance. The TAM model theorizes that beliefs about perceived ease of use and perceived usefulness are the main constructs predicting user intention. In recent years, this model has been extended and modified in a dozen studies. One of the extended TAM models is the unified theory of acceptance and use of technology (UTAUT) [12]. Besides ease of use (in this model called “effort expectancy”) and perceived usefulness (called “performance expectancy”), 2 other key constructs are added in the UTAUT model: social influence and facilitating conditions. In addition, gender, age, experience, and voluntariness of use are included in this model as moderators that influence the key constructs on intention to use. The TAM and UTAUT models have been frequently applied in health care research [13]. However, they are not often utilized to investigate patient acceptance of eHealth services [10]. These TAMs are constantly evolving. Or and Karsh [10] suggested in their review that, besides the before mentioned constructs, the influence of trust on patients’ acceptance should be further explored, because trust is found to be a predictor of technology acceptance in research outside the field of health care. In addition, attitude is not a direct determinant in the original UTAUT model of Venkatesh [12]. However, several studies suggest that there is a relation between attitude and intention, for example [14].

The primary objective of this study is to investigate the actual use and intention toward using Internet services to communicate with the general practice by the general practice population. The secondary objective is to get insight into characteristics and factors that influence the intention to use such services by the general practice population. The goal of the study was not to develop and validate a new model to predict patients’ intention to use Internet communication services. For the secondary objective, 2 services are specifically studied: making an appointment on the Internet (related to the Internet accessibility of the general practice) and asking a question via email or a website (related to gathering information about health content on the Internet). These services are relatively easy to access, but can have a major impact on daily care routines. The focus was on these 2 services because many general practices already offer them to their patients or intend to implement these services in the near future.

## Methods

### Design and Participants

There were 1500 participants of the Dutch Health Care Consumer Panel [15], aged over 18 years, who were invited to take part in this cross-sectional study. This panel was established by the Netherlands Institute for Health Services Research (NIVEL) and the Dutch Consumer Association. The sample was representative of the Dutch population in terms of age and gender based on data of Statistics Netherlands [16]. People who contacted the GP at least once in the past year were included in this study. Questionnaires were used for data collection. The panel members could choose whether they wanted to receive a questionnaire by post or email. The questionnaires were issued in April 2013.

### Measurements

#### Participant Characteristics

The background characteristics of the members of the health care consumer panel had already been gathered using a questionnaire that was completed at the start of their membership. For this study, the following characteristics were used: gender, age, level of education, and whether they had none or at least one chronic disease. Furthermore, participants indicated whether they rated Internet use as easy or difficult on a 5-point Likert scale, ranging from 1 (very difficult) to 5 (very easy). In addition, they could indicate that they did not use the Internet.

#### Use, Intention to Use, and Availability of Internet Services

Participants were asked to fill out a questionnaire regarding the use of the following 6 Internet services to communicate with the general practice: (1) Internet appointment planning, (2) asking questions by Internet via email or a website, (3) email reminders about appointments, (4) Internet prescription refill requests, (5) Internet access to medical data, and (6) Internet video consultation. Participants were asked to indicate whether they had used these services in the past year. If they had not used the service in the past year, the participants were asked about their intention toward using the service (either positive or negative intention). They could also indicate that they did not know whether they would like to use the service. Furthermore, the participants indicated whether they thought these services were available at their general practice or not, or that they did not know whether this service was available.

#### Factors Influencing Intention to Use Internet Services

To study which factors influence the intention to use Internet appointment planning and the asking of questions by Internet via email or a website, participants rated items on a 4-point Likert scale, ranging from 1 (strongly disagree) to 4 (strongly agree). For these questions, the option of “don’t know” was added. For both services, participants rated items that were divided according to the following 6 subscales: effort expectancy (2 items), trust (2 items), attitude (1 item), facilitating conditions (1 item), social influence (1 item), and performance expectancy (3 items). For the service of asking questions via email or a website, 2 items were added to the performance expectancy scale. The items regarding effort expectancy, facilitating conditions, social influence, and performance expectancy were mainly based on the validated UTAUT model [12], as well as on recommendations of studies by Or and Karsh (trust) [10] and Spil and Schuring (attitude) [14]. First, the items of the 6 subscales were asked for Internet appointment planning, and, subsequently, for the service of asking questions by Internet. Participants’ mean scores on each subscale were calculated. A list of all the items is presented in Appendix 1 (see Multimedia Appendix 1).

### Statistical Analyses

Descriptive analyses were conducted to study participant characteristics and to investigate participants’ actual use, intention toward using, and awareness of availability regarding the 6 Internet services. Only participants who filled out all items regarding each of these were included in the analyses. The outcomes are expressed in percentages or in means and SDs.

Linear correlation analyses were conducted to identify multicollinearity in the 6 constructs of effort expectancy, trust, attitude, facilitating conditions, social influence, and performance expectancy for the 2 Internet services: Internet appointment planning and asking questions via email or a website. Items based on the UTAUT model, which were scored as “don’t know,” were analyzed as missing data. In addition, variance inflation factors (VIFs) were calculated to assess multicollinearity. Correlation coefficients above .8 were considered high, and VIF values above 10 [17] were considered to be unacceptable. Therefore, constructs with a VIF value above 10 were left out of further analyses.

To test which characteristics and factors influence participants’ intention toward using the 2 services, univariate logistic regression analyses were conducted. In these analyses, intention to use (1=users + nonusers with a positive intention, 0=nonusers with a negative intention) was the dependent variable. For each of the 2 services, 6 univariate logistic regression analyses were conducted with the mean scores of the following subscales as independent variables: effort expectancy, performance expectancy, trust, attitude, facilitating conditions, and social influence. In addition, 5 univariate logistic regression analyses were conducted with the following characteristics as independent variables: gender (1 = male, 0 = female), age (1 = ≥65 years, 0 = <65 years), chronic condition (1 = at least one, 0 = none), level of education (low, middle, and high), and Internet usage (1 = easy and very easy, 0 = nonuser, very difficult, difficult, and neutral). Outcomes were expressed in odds ratios (OR) and 95% confidence intervals (CI). Bonferroni correction is applied to reduce the bias of multiple testing. All effects are reported at a *P*=.005

## Results

### Participants

**Figure 1:**
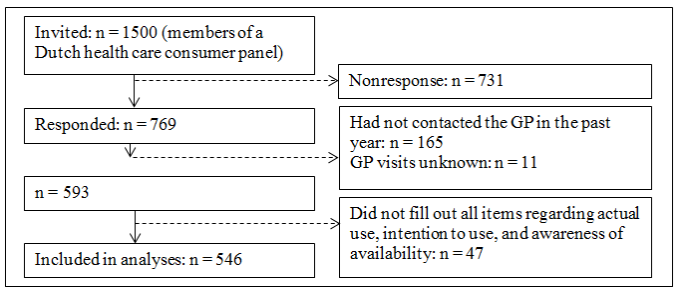
Flowchart of participants included in the study. GP: general practitioner.

Figure 1 shows a flowchart of the process of the inclusion of participants. Out of 1500 participants, 769 responded to the questionnaire (63.3%, 487, of these participants responded by Internet). Of these participants, 176 were excluded because they had not contacted their GP in the past year (n=165) or did not respond to the question concerning GP visits (n=11). Furthermore, participants were excluded from further analyses if they did not fill out all items regarding actual use, intention toward using, and awareness of availability of all 6 services (n=47). This resulted in a total sample of 546 participants. Table 1 shows the characteristics of the study sample.

**Table 1:** Characteristics of the study sample (n=546).

Characteristics		Mean (SD) or n (%)
Age in years		53.14 (15.4)
**Gender**		
	Men	238 (43.6)
	Women	308 (56.4)
**Level of education**		
	Low	69 (12.6)
	Medium	306 (56.0)
	High	156 (28.6)
	Unknown	15 (2.8)
**Chronic condition (self-reported)**		
	None	132 (24.2)
	At least one	365 (66.8)
	Unknown	49 (9.0)
**Internet usage**		
	No internet	35 (6.4)
	(Very) difficult or neutral (score 1, 2, 3)	141 (25.8)
	(Very) easy (score 4, 5)	352 (64.5)
	Unknown	18 (3.3)
**Data collection**		
	By post	189 (34.6)
	By internet	357 (65.4)

### Use, Intention to Use, and Awareness of Availability of Internet Services

**Figure 2:**
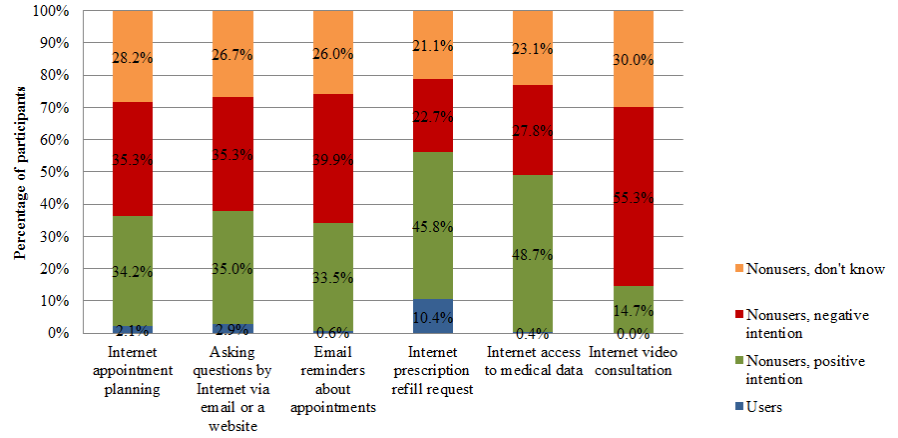
Percentage of participants who had used the Internet care service in the past year, and participants’ intention toward the use of the Internet services.

Overall, the actual usage of Internet services to communicate with the general practice is low. Not one of the participants had an Internet video consultation with the GP in the past year, 0.4% (2/546) had Internet access to their medical data, 0.6% (3/546) received email reminders about appointments, 2.2% (12/546) planned an appointment by Internet, and 2.9% (16/546) asked a question via email or a website. Requesting a prescription refill by Internet was the most frequently used service (10.4%, 57/546). Figure 2 shows an overview of the results.

Participants who had not used the Internet service in the past year could indicate whether they would like to use the service in the future. These results are also presented in Figure 2. The percentage of participants who had a positive intention toward Internet video consultation was 14.7% (80/546). Approximately one third of the participants had a positive intention toward receiving email reminders about appointments (33.5%, 183/546), Internet appointment planning (34.2%, 187/546), and asking questions via email or a website (35.0%, 191/546). The highest percentages of participants with a positive intention were found for Internet prescription refill requests (45.8%, 250/546) and having access to medical data (48.7%, 266/546). The percentage of participants with a negative intention varied between 22.7% (124/546, Internet prescription refill requests) and 55.3% (302/546, Internet video consultation). For each service, more than one fifth of the participants responded that they did not know whether they would like to use the Internet service, ranging from 21.1% (115/546, Internet prescription refill requests) to 30.0% (164/546, Internet video consultation).

**Figure 3:**
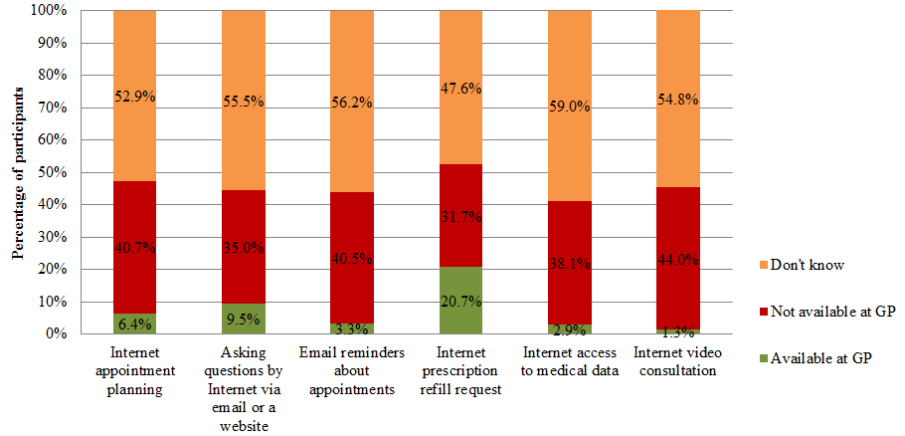
Participants’ awareness of the availability of Internet care services at their primary care practice. GP: general practitioner.

Figure 3 shows the percentage of people who either knew or did not know whether each of the Internet services was available at their general practice. There were 1.3% (7/546) of the participants who responded that Internet video consultation was possible at their GP, and 20.7% (113/546) responded that requesting a prescription refill by Internet was possible. However, those who indicated that Internet services were not available at their general practice ranged from 31.7% (173/546) of the sample, who indicated that requesting prescription refills by Internet was not available, to 44.0% (240/546), who indicated that Internet video consultation was not available. In addition, for each Internet service, approximately half of the participants did not know whether the service was available at their primary care center.

### Associations Between Factors and Intention to Use Internet Services

Tables 2 and 3 show the correlation matrices of the constructs (effort expectancy, performance expectancy, trust, attitude, facilitating conditions, and social influence). The number of participants included in separate correlation analyses differs, due to many “don’t know” responses to items. There were 115 participants who answered all the items (n=10) regarding the constructs that can influence intention to use Internet appointment planning, without using the “don’t know” option; 94 participants did this regarding asking questions by Internet via email or a website (12 items). The correlations between all constructs were statistically significant and higher than or equal to *r*=.45 (*P*<.001) for both services. Of the correlation coefficients between the independent constructs that could influence Internet appointment planning, 6 correlation coefficients exceeded the value of .80, which is considered to be high: trust was related to effort expectancy (*r*=.82), attitude (*r*=.81), and social influence (*r*=.81); and attitude was related to facilitating conditions (*r*=.85) and social influence (*r*=.86). VIFs were calculated to identify the extent to which the constructs were interrelated. Not one of the VIF values exceeded the cutoff point of 10, indicating that the assumption of multicollinearity was not violated. For constructs influencing the intention toward using a service to ask questions via email or website, 2 correlation coefficients were found which exceeded the value of .80: trust was related with effort expectancy (*r*=.86) and facilitating conditions (*r*=.85). In addition, the VIF value for trust was 12.92, which exceeds the cutoff point. Therefore, the construct trust was left out of the univariate logistic regression analysis.

**Table 2:** Matrix of linear correlations and variance inflation factor values between the independent constructs that could influence intention to use Internet appointment planning.^a^

	EE^b^	PE^c^	TR^d^	AT^e^	FC^f^	VIF value^g^
1. EE						7.25
2. PE	.45 n=314					1.75
3. TR	.82 n=263	.56 n=259				7.08
4. AT	.71 n=283	.61 n=312	.81 n=249			8.84
5. FC	.73 n=314	.53 n=347	.75 n=261	.85 n=308		4.56
6. SI^h^	.78 n=162	.63 n=186	.81 n=154	.86 n=167	.80 n=176	4.05

^a^All results are found to be significant at the *P*<.01 level.

^b^EE: effort expectancy

^c^PE: performance expectancy

^d^TR: trust

^e^AT: attitude

^f^FC: facilitating conditions

^g^VIF: variance inflation factor

^h^SI: social influence

**Table 3:** Matrix of linear correlations and variance inflation factor values between the independent constructs that could influence intention to ask questions by Internet via email or a website.^a^

	EE^b^	PE^c^	TR^d^	AT^e^	FC^f^	VIF value^g^
1. EE						7.35
2. PE	.56 n=307					2.93
3. TR	.86 n=244	.63 n=247				12.92
4. AT	.60 n=259	.69 n=307	.77 n=221			4.42
5. FC	.79 n=287	.64 n=319	.85 n=237	.70 n=279		6.98
6. SI^h^	.73 n=142	.76 n=158	.80 n=132	.78 n=144	.74 n=142	4.65

^a^All results are found to be significant at the *P*<.01 level.

^b^EE: effort expectancy

^c^PE: performance expectancy

^d^TR: trust

^e^AT: attitude

^f^FC: facilitating conditions

^g^VIF: variance inflation factor

^h^SI: social influence

Table 4 shows the results of the univariate logistic regression analyses. All constructs (effort expectancy, performance expectancy, trust, attitude, facilitating conditions, and social influence) had a significant association with intention to use Internet appointment planning and asking questions via email or a website. For Internet appointment planning, the ORs varied between 3.28 (95% CI 2.21-4.86) for effort expectancy and 8.51 (95% CI 5.15-8.51) for attitude. For asking questions via email or a website, the ORs varied between 5.46 (95% CI 4.34-7.86) for social influence and 7.91 (95% CI 4.53-13.82) for facilitating conditions.

**Table 4:** Univariate association of constructs and characteristics with intention toward using Internet appointment planning and asking questions by Internet via email or a website. All constructs and characteristics had a significant association with intention to use both services, except for gender and chronic condition.

Independent variable	Dependent variable: intention to use Internet appointment planning		Dependent variable: intention to use a service to ask questions by Internet via email or a website	
	n	Odds ratio (95% CI)	n	Odds ratio (95% CI)
Perceived ease of use	264	3.28 (2.21-4.86)	252	5.46 (3.27-9.13)
Perceived usefulness	301	3.98 (2.58-6.14)	284	5.47 (3.44-8.70)
Trust	226	5.16 (3.21-8.15)	—	—
Attitude	263	8.51 (5.15-14.07)	238	5.85 (3.63-9.43)
Facilitating conditions	283	5.32 (3.51-8.08)	254	7.91 (4.53-13.82)
Social influence	150	4.80 (2.83-8.16)	119	4.34 (2.46-7.68)
Gender	392	0.90 (0.06-1.33)	400	0.92 (.62-1.37)
Age	392	0.172 (0.10-.29)	400	0.14 (.084-.24)
Level of education	380	2.53 (1.78-3.60)	387	2.24 (1.58-3.17)
Chronic condition	357	0.79 (0.49-1.26)	361	0.71 (.44-1.14)
Internet usage	381	7.98 (4.74-13.44)	389	7.97 (4.97-13.23)

Looking into characteristics of participants, age, level of education, and Internet usage had a significant association with intention to use Internet appointment planning and asking questions via email or a website. The ORs for age were 0.172 (95% CI 0.10-0.29) and 0.14 (95% CI 0.084-0.24), respectively. The ORs for level of education were 2.53 (95% CI 1.78-3.60) and 2.24 (95% CI 1.58-3.17), respectively. ORs for Internet usage were 7.98 (95% CI 4.74-13.44) and 7.97 (95% CI 4.97-13.23), respectively.

## Discussion

### Principal Results and Comparison With Previous Work

This study indicates that Internet communication services used for contacting the general practice by the general practice population are not yet frequently used in the Netherlands. Of the participants who had not used the service in the previous year, the percentage of participants with a positive intention toward using a service varied between approximately 15% (Internet video consultation) and approximately 5% (having access to medical data). Many participants were not aware of the availability of such services at their general practice, as approximately half of the participants did not know whether such a service was available at their primary care center. Possible factors and characteristics that influence intention to use Internet communication services were investigated in this study. Univariate logistic regression analyses revealed that the following constructs had a significant influence on intention to use Internet appointment planning and asking questions via email or a website: effort expectancy, performance expectancy, trust, attitude, facilitating conditions, social influence, the characteristics of age, level of education, and Internet usage. However, many participants responded with “don’t know” to items regarding intention. In addition, high correlations are found between the constructs. This indicates that the Dutch population has no strong view regarding the use and possibilities of Internet services for communicating with the general practice.

In this study, it is found that the use of the Internet to communicate with the general practice is still low. This is in line with findings of previous research [9,18-20]. Although the actual use of such Internet services is low, the Internet is frequently used for health purposes in Europe [18]. It is even the main source of health-related information for the Dutch population [21]. Access to the Internet and the availability of Internet communication services are the key preconditions for successful uptake and usage of Internet services. These conditions seem to be promising in the Netherlands: 94% of households have access to the Internet at home, and 55% of people between 65 and 75 years of age access the Internet almost every day [16]. In addition, more than 90% of GPs offer Internet communication services to their patients [3]. One of the reasons that the actual use of these services is not as high as expected might be that the general practice population is not aware of the availability of the Internet services offered by their primary care practice. In this study, less than 20.7% (113/546) of participants indicated that an Internet service was available at their general practice. Moreover, at least 47.6% (260/546) of the study sample did not know if an Internet service was available at their primary care practice. This is in contrast with the high number of primary care practices that offer such services [3]. Our study confirms the findings of previous research, which has concluded that often patients do not know about the existence of eHealth applications or they are not aware of the possibilities of the applications [22]. Moreover, Mair et al [23] concluded in their review of factors that promote or inhibit the implementation of eHealth services that specifying the purposes, benefits, and values of eHealth services to users during the implementation (the “sense-making” process) is not well covered in previous studies. The fact that the general practice population is not well informed about the availability and possibilities of Internet services might explain the high number of “don’t know” responses in our study.

Effort expectancy, performance expectancy, trust, attitude, facilitating conditions, and social influence are found to be constructs that influence the intention to use Internet communication services by the general practice population. However, in looking into the relationships between the independent constructs in this study, moderate to high correlations were found. Although the assumption of multicollinearity is only violated for 1 variable (trust), it should be questioned whether these subscales measure different constructs. Although the UTAUT model is frequently applied in health research [13], it is not yet frequently used to investigate patients’ intention toward using Internet services in health care [10]. In the few studies that have applied (a modified version of) this model to predict patient acceptance of Internet services that support self-management, high correlations between the independent constructs were either not reported [24,25] or not found [26]. Furthermore, in studies that have applied the UTAUT model to examine health care professionals’ acceptance of eHealth services, low to moderate correlations between constructs have been found, for example [27,28]. It might be the case that Internet services for communicating with the general practice is not a major issue in Dutch society and therefore participants had no strong opinion about these services. Further research is recommended to investigate whether the UTAUT model is applicable for the investigation of intention to use Internet communication services by the general practice population.

The influence of patient characteristics on intention to use eHealth services is well studied [10]. In this study, an older age, lower level of education, and the rating of Internet usage as difficult, is associated with a negative intention. This is in line with most, but not all, previous research which is studied in the review by Or and Karsh [10]. Some researchers argue that the negative association between age and information and communication technology (ICT) usage will disappear within a few years as the older generation become more familiar with using it; however, a recent study by Heart and Kalderon [29] found that although there is an increase in ICT adoption among older people, they are not yet ready to adopt health-related ICT. In their study, “no need” to use ICT was found to be the most prevalent reason for nonuse, and therefore, it is suggested that health care providers should clearly demonstrate the benefits of Internet services to their customers. In this study, no association between gender and intention to use Internet communication services in primary care was found, which corroborates most previous studies [10]. Having no, or at least one, chronic condition was not associated with intention to use. The effect of patient health status on the use of eHealth services has yielded mixed results in previous research [10]: some studies have found no association between these constructs, for example [30], whereas others have found increased acceptance in people with a better, for example [31], or a poorer health status, for example [32,33]. Furthermore, Heart and Kalderon [29] found that health status moderated the effect of age on use. In this study, participants could indicate their chronic conditions using a questionnaire. However, having one or more chronic condition(s) does not automatically result in different health-seeking behaviors. The number of general practice visits might indicate this better. Future research is recommended to investigate whether this has an influence on intention to use Internet communication services in primary care.

### Strengths and Limitations

A strength of this study is that it aimed to investigate the actual use and intention to use Internet communication services, which are currently being implemented in primary care practices. A high number of participants (n=546) between 18 and 83 years of age participated in this study. However, this was not a representative sample of the actual Dutch patient population, which visits the GP at least once a year [34]. There was an underrepresentation of elderly people, which could have led to an overestimation of the intention to use Internet services, because age is found to be associated with intention to use.

Another limitation of this study is that participants who actually used an Internet service were not asked whether they had a positive or negative intention toward using the service in the future. However, because they should have had a positive intention toward using it in the past, these participants were analyzed as having a positive intention. In addition, the true availability of the Internet communication services was not investigated in this study. While the overall percentage of primary care practices that offer such services is known, it is not known whether these services were also available for participants of this study.

The main content of the questionnaire to investigate intention to use the service of Internet appointment planning and asking questions by Internet is based on the validated UTAUT model [12]. The subscales of trust and attitude are not validated. However, the goal of the secondary objective was not to develop a new validated model that predicts patients’ intention to use Internet care services. In addition, it is not claimed that the included factors are the only predictors of intention to use Internet care services. The goal was to get insight into possible predictors of intention to use Internet communication services by the general practice population by applying suggested predictors found in literature.

Participants could choose to receive the questionnaire on paper or via the Internet. The use of a mixed data collection methodology could be seen as a limitation of this study. However, based on previous research, it is not expected that this significantly influenced the results [35,36]. In addition, by giving the participants the choice to fill out the questionnaire on paper or via the Internet, a broader study sample was covered.

No multivariate logistic regression analyses could be performed, due to too many “don’t know” responses to items that could influence intention to use Internet communication services in primary care, which were analyzed as missing data. Therefore, it could not be indicated which of the studied constructs has the strongest association with intention to use. Moreover, due to the fact that “don’t know” responses were analyzed as missing data, the studied sample only consists of people who actually had an opinion (positive or negative) about the Internet communication services. This could have led to a misrepresentation of the sample. An alternative option for dealing with missing data due to “don’t know” responses is to impute the mean score of a subscale to the missing value of that subscale. However, this method could not be applied because 3 subscales consisted of 1 item and many participants filled out “don’t know” to all items in 1 subscale. Another option is imputing a neutral response (score 2.5) for missing data. Although the authors believe that this is not the same as “don’t know,” repeating the univariate analyses with this response option did not change the results. In addition, the high number of “don’t know” responses to the items suggests that people have difficulties in evaluating their expectations of the use of Internet communication services in primary care. By giving them the option of “don’t know,” they were not forced to choose between agree and disagree, resulting in a more reliable set of responses.

### Conclusions

This study has found that Internet communication services to contact the general practice are not yet frequently used by the general practice population. Many participants indicated that they did not know whether such a service was available at their primary care center. In addition, although a substantial number of people had a positive intention toward using such services, the entire general practice population did not seem willing to use them. Informing the general practice population about the availability and possibility of such services during their implementation might be important for stimulating the uptake and usage of Internet communication services in primary care.

## Appendix

Multimedia Appendix 1 Items related to the constructs that influence intention to use Internet services, rated on a four-point Likert scale ranging from 1 (strongly disagree) to 4 (strongly agree).

## Abbreviations

CI: confidence interval
GP: general practitioner
ICT: Information and communication technology
NIVEL: Netherlands Institute for Health Services Research
OR: odds ratio
TAM: technology acceptance model
UTAUT: unified theory of acceptance and use of technology
VIF: variance inflation factor
