# Supplementary material for: Internet Services for Communicating With the General Practice: Barely Noticed and Used by Patients
Source: Interact J Med Res. 2015 Nov 24;4(4):e21. doi: 10.2196/ijmr.4245 (PMC4704911; doi:10.2196/ijmr.4245)
Supplement: Multimedia Appendix 1 [file ijmr_v4i4e21_app1.pdf]

Items related to the constructs that influence intention to use Internet services, rated on a four-point Likert scale ranging from 1 (strongly disagree) to 4 (strongly agree).

Making an Internet appointment /asking a question by Internet via email or a website is:

Effort expectancy
1. Easy to use
2. Easy to learn

Trust
3. Is reliable
4. Works properly

Attitude
5. Is a pleasant way

Facilitating conditions
6. Is easy to combine with my daily activities

Social influence
7. Is something that my family/friends would like to use

Performance expectancy
8. Ensures that I can make an appointment/ask a question more easily
9. Ensures that I can make an appointment/ask a question more often
10. Ensures that I can decide myself if an appointment is necessary [a]
11. Ensures that I can easily ask questions about a given answer [b]
12. Ensures that I can properly think about a question I would like to ask [b]
13. Ensures that I get more personal attention from my GP [b]

[a] Only asked for the service of Internet appointment planning.
[b] Only asked for the service of asking questions by Internet via email or a website.
